# Mitochondrial medicine in obesity: a scoping review

**DOI:** 10.1515/med-2026-1407

**Published:** 2026-04-08

**Authors:** Amedeo Lonardo, Ralf Weiskirchen

**Affiliations:** AOU Modena, Ospedale Civile di Baggiovara (-2023), Modena, Italy; Institute of Molecular Pathobiochemistry, Experimental Gene Therapy, and Clinical Chemistry (IFMPEGKC), RWTH University Hospital Aachen, Aachen, Germany

**Keywords:** adipose tissue, kidneys, female fertility, muscle, steatotic liver disease, pancreas

## Abstract

**Introduction:**

Mitochondrial dysfunction connects obesity and metabolic dysfunction. We conducted a scoping review on mitochondria in obesity to (i) describe morpho-functional mitochondrial abnormalities across tissues and (ii) summarize mitochondria-directed lifestyle and pharmacological strategies and their metabolic effects.

**Content:**

PubMed and Web of Science were searched, using relevant keywords. English-language original studies, clinical trials, and systematic reviews were qualitatively synthesized in a scoping review conducted in accordance with the PRISMA extension for Scoping Reviews and prospectively registered in the Open Science Framework.

**Summary:**

Sixty primary articles and cross-references describe nutrient overload-induced oxidative stress, disturbed mitochondrial dynamics and mitophagy, and maladaptive endoplasmic reticulum (ER)-mitochondria contacts across various organ tissues and cancer. These changes are associated with insulin resistance, steatotic liver disease, chronic kidney disease, cardiovascular dysfunction, impaired fertility, and tumor progression. Reported interventions include mitochondria-targeted antioxidants, AMPK/SIRT1/PGC-1α activators, modulators of ER-mitochondria coupling, microbiota-directed approaches, and lifestyle changes. Common mitochondrial signatures, excess reactive species, impaired quality control, and altered organelle crosstalk, underlie systemic metabolic derangement, supporting mitochondria as a unifying therapeutic target.

**Outlook:**

Obesity involves widespread mitochondrial changes in various organs. Approaches that improve mitochondrial health through lifestyle and medication may help manage obesity complications and need thorough clinical testing.

## Background and aims

The global prevalence of obesity is one of the most serious public health challenges of the 21st century. According to the World Obesity Federation, the United Nations, and the World Health Organization (WHO) its prevalence has tripled since 1975 and now affects over 650 million adults, 340 million adolescents, and 39 million children worldwide [1], 2]. Far from being a passive energy store, adipose expansion triggers a cascade of systemic physiopathological consequences including hyperlipidemia, lipotoxicity, low-grade inflammation, and insulin resistance that all converge on the mitochondrion, the main bioenergetic and metabolic hub of the cell and key players in preserving cellular homeostasis and signaling [3], [4], [5]. Compelling evidence shows that excess nutrients lead to the generation of reactive oxygen species (ROS) and nitrogen species (RNS) in mitochondria, causing oxidative damage to electron-transport chain (ETC) complexes, disruption of mitochondrial dynamics, impaired fatty acid β-oxidation, and faulty mitophagy, all contributing to metabolic decline [6], 7]. Recent high-resolution volumetric electron-microscopy studies now reveal that these functional abnormalities are linked to significant architectural changes: in lean fasted mice, mitochondria enlarge and form adaptive connections with rough endoplasmic reticulum (ER) sheets through the tethering protein Ribosome-binding protein 1 (RRBP1), while in obesity, this structural flexibility is diminished and replaced by an excess of tight smooth-ER/mitochondria contact sites, known as mitochondria-associated membranes (MAMs) that enhance maladaptive Ca^2+^ and lipid signaling [8], 9]. Multifactorial determinants regulate microbiota composition including resistance to stress, presence of pathogens, diet, cultural habits and general health conditions [10]. At the same time the gut microbiota regulates gut microbiota-derived metabolites (like secondary bile acids and short-chain fatty acids), crucial transcription factors, coactivators, as well as enzymes implicated in influencing mitochondrial biogenesis, metabolism, autophagosome formation and oxidative stress responses, thus regulating the activation of the inflammasome and the production of inflammatory cytokines, key players in chronic metabolic disorders [10], [11], [12]. Mitochondrial ROS, in turn, impact intestinal barrier integrity and microbial composition, positioning mitochondria at the core of a bidirectional two-way host-microbe axis in metabolic regulation [10], [11], [12].

These discoveries have sparked significant interest in “mitochondrial medicine,” a rapidly growing field that includes mitochondria-targeted antioxidants such as the triphenyl phosphonium-ubiquinone conjugate MitoQ or mitochondria-targeted peptides with antioxidant activity known as Szeto–Schiller peptides [13], [14], [15], [16]. Additionally, there are enhancers of mitophagy and biogenesis (e.g., AMPK/SIRT1/PGC-1α activators), modulators of ER-mitochondria connections, and microbiota-directed strategies like probiotics, prebiotics, and fecal microbiota transplantation [13], [14], [15], [16]. However, the translation of these approaches to clinical practice is still in its early stages, hindered by diverse methodologies, varying endpoints, and an incomplete comprehension of organelle-centric mechanisms across different tissues and metabolic conditions.

Based on the grounds described above, the objectives of this scoping review are three-fold:

1. To outline the range of mitochondrial structural and functional abnormalities that characterize obesity, integrating recent ultra-structural, biochemical and omics-level findings.
2. To critically evaluate current therapeutic strategies aimed at restoring mitochondrial health, including pharmacological agents, lifestyle interventions and microbiota-based modalities, and assess their efficacy in pre-clinical and clinical settings.
3. To identify existing knowledge gaps and suggest future research priorities for utilizing mitochondrial biology in the prevention and treatment of obesity and its metabolic complications.

Given the breadth of the topic and the heterogeneity of study designs, populations, and mitochondrial endpoints, we chose a scoping review design to map key concepts, summarize the extent and nature of the evidence, and identify gaps rather than to perform a quantitative meta-analysis. Accordingly, we structured the review and its reporting following the PRISMA extension for Scoping Reviews (PRISMA-ScR), proposed by Tricco and colleagues [17].

## Methods

We conducted a systematic search of PubMed and Web of Science using a combination of controlled vocabulary and free-text words related to “obesity,” “mitochondria,” “mitochondrial dysfunction,” “mitochondrial therapy,” “mitochondria-targeted antioxidant,” “mitophagy,” “biogenesis,” and “metabolic disease.” The methodological conduct and reporting of this review were guided by recommendations for scoping reviews and adhered to the PRISMA-ScR reporting checklist. Eligible for inclusion were original research articles, systematic reviews and clinical trials published in English. We screened titles, abstracts, and full texts to extract data on study design, population, intervention, mitochondrial endpoints (such as respiration, ROS, dynamics, and organelle contacts), and metabolic outcomes (such as insulin sensitivity, lipid profiles, and weight). The completed PRISMA-ScR checklist is provided in Supplementary Table S1.

## Results

### Search and selection of sources of evidence

Our initial search in PubMed and Web of Science, using the Title keywords “Obesity” AND “Mitochondria” from inception through 10 November 2025, retrieved 72 articles. 12 of these records (listed in the Supplementary References) were excluded because they were editorials, reviews, not directly relevant, or considered outdated. The remaining 60 primary articles, together with cross-references and additional articles previously published by the present authors, formed the evidence base on which this scoping review was developed. Due to heterogeneity in study designs and outcome measures, we conducted a qualitative synthesis. We initially adopted a restrictive search strategy to guarantee specificity in a field with an extremely large number of publications, and subsequently broadened the evidence base by examining cross-references from the selected articles.

### Study registration

The review protocol was prospectively registered in the Open Science Framework (OSF) as a scoping review under registration ID: https://doi.org/10.1515/med-2026-1407.

## Phylogenesis, normal structure, and physiological energetic and signaling functions of mitochondria

### Normal structure and physiological functions of mitochondria

Mitochondria are dynamic, double-membrane organelles present in virtually every eukaryotic cell, with densities and ultrastructure tailored to tissue-specific energetic needs [18]. All mitochondria originate from a common ancestor: an alphaproteobacterium that became endosymbiotic within a host related to *Asgard Archaea* [19]. Transitioning from bacterium to organelle required extensive evolutionary changes, which occurred gradually as host and endosymbiont integrated. Despite many advances in understanding this process, debates remain about the endosymbiont’s nature, its early relationship with the host, and its timing relative to other eukaryotic features. Since then, mitochondrial proteins have been gained, lost, or transferred as mitochondria diversified across eukaryotes [19].

The smooth outer mitochondrial membrane (OMM), studded with voltage-dependent anion channels (VDACs), is freely permeable to metabolites up to approximately 5 kDa. In contrast, the highly invaginated inner mitochondrial membrane (IMM) contains five protein complexes, including the electron-transport chain (complexes I–IV) and adenosine triphosphate (ATP) synthase (complex V) [20], 21]. Proton pumping across the IMM serves as the major source of cellular energy by establishing the electrochemical gradient mitochondrial membrane potential (Δψm) that drives oxidative phosphorylation (OXPHOS), yielding the bulk of cellular ATP. The matrix accommodates the tricarboxylic acid (TCA) cycle, fatty-acid β-oxidation spiral, amino-acid catabolism, and a portion of the urea cycle, thereby integrating carbon and nitrogen flux with energy production [22]. Mitochondria also synthesize haem, Fe–S clusters, cardiolipin and steroid hormones, underscoring their biosynthetic versatility [23], 24].

Physiologically generated ROS-, including superoxide (O^2−^), hydrogen peroxide (H_2_O_2_) and, through reaction with nitric oxide, peroxynitrite (ONOO^−^), serve as redox messengers that modulate insulin receptor signaling, hypoxia responses and autophagy when maintained at low levels [25], 26] Antioxidant defenses (SOD2, GPx, catalase, thioredoxin, the NRF2–KEAP1 system) normally keep ROS in check [27], 28]. However, when nutrient oversupply or cytokine exposure overwhelms these defenses, Δψm collapses, ETC electron transport reverses, and oxidative/nitrosative stress propagates damage to proteins, lipids and mitochondrial DNA (mtDNA) [29], 30]. Damaged or depolarized mitochondria are selectively removed by mitophagy, a quality-control pathway initiated by PINK1-mediated recruitment of the E3 ligase Parkin and reinforced by BCL2/BNIP3-family adaptors that tether defective organelles to microtubule-associated proteins 1A/1B light-chain 3 (LC3)-decorated autophagosomes [31], [32], [33].

Mitochondria do not exist as isolated spheres, but as highly interconnected tubulovesicular networks whose shape is determined by the balance of fusion (MFN1/2, OPA1) and fission (DRP1, Fis1) events [34], 35]. Fusion allows for content mixing, optimizes respiratory efficiency and buffers stress, while fission segregates dysfunctional segments for elimination or facilitates redistribution to areas of high energy demand. Recent volume electron-microscopy has revealed further layers of architectural complexity: in hepatocytes of fasted lean mice, mitochondria expand and form quasi-continuous contacts with single rough ER sheets, a configuration dependent on the ribosome-binding protein RRBP1. These “wrapper” interactions are spatially enriched in periportal and mid-lobular zones where fatty-acid oxidation predominates [8]. Conversely, mitochondria also make tight (10–25 nm) contacts with smooth ER tubules at MAMs, which are hubs for Ca^2+^ flux, lipid transfer, and inflammasome activation [36], 37].

These structural arrangements enable mitochondria to fulfil additional signaling roles. Ca^2+^ uptake through the mitochondrial calcium uniporter couples cytosolic Ca^2+^ transients to ATP production, while the release of metabolites and ROS modulates nuclear transcription factors such as NRF2 and NF-κB, influencing antioxidant, inflammatory and metabolic gene programs [38], [39], [40]. In the liver, zonated heterogeneity means periportal mitochondria display higher oxidative capacity and oxygen availability than pericentral counterparts; fasting shifts mitochondrial size, shape and ER contacts preferentially in periportal and mid-lobular hepatocytes, illustrating how subcellular architecture underpins metabolic flexibility [8]. Maintaining this finely tuned mitochondrial structure-function axis is essential for systemic energy homeostasis, a principle that forms the rationale for targeting mitochondria in the treatment of obesity and related metabolic diseases [41], 42].

### Alterations in mitochondrial functions during obesity

Obesity results in an excess of nutrients and inflammatory mediators that disrupt mitochondrial bioenergetics, redox balance and quality-control pathways. In adipose tissue (AT) and the liver, an abundance of circulating free fatty acids overwhelms the oxidative capacity of the mitochondria, leading to a buildup of incomplete β-oxidation products, ETC overload, and increased electron leak [43]. This causes elevated levels of superoxide, hydrogen peroxide, and peroxynitrite, surpassing the antioxidative defense systems (such as SOD2, GPx, catalase) and shifting the redox balance towards persistent oxidative/nitrosative stress [27], 44], 45]. As a result, oxidative damage to ETC complexes I, III, and IV occurs, reducing ATP production, worsening mitochondrial dysfunction and impairing insulin-signaling [46], 47].

Obesity also disrupts the normal structure of the mitochondrial network. In obese individuals, the expression of the fusion mediator MFN2 is down-regulated in skeletal muscle and hepatocytes, while dynamin-related protein 1 (DRP1)-dependent fission is stimulated. This leads to fragmented, poorly interconnected organelles with reduced cristae density and impaired substrate transport [48]. Studies using high-resolution electron microscopy have shown that in obese livers, mitochondria do not adaptively enlarge and elongate during fasting, remaining small, round, and pushed towards the cell periphery by lipid-droplet expansion, indicating a loss of architectural flexibility and metabolic adaptability [8].

Quality-control mechanisms are also affected by obesity. Excess ROS causes damage to cardiolipin and mtDNA, triggering PTEN-induced kinase 1 (PINK1)/Parkin‐mediated mitophagy. However, chronic nutrient stress impairs the formation of autophagosomes and lysosomal turnover, allowing damaged mitochondria to accumulate [49]. This mitochondrial distress activates the NLR family pyrin domain containing 3 (NLRP3) inflammasome through ROS- and TXNIP-dependent pathways, leading to the release of IL-1β and IL-18, which worsens insulin resistance in the liver and AT [50].

Inter-organelle communication is selectively rewired. Obesity significantly increases tight contacts (10–24 nm) between smooth ER tubules and mitochondria (known as the MAMs), while reducing the formation of adaptive rough-ER sheet-mitochondria interactions that typically expand during fasting. Enhanced MAM tethering intensifies Ca^2+^ transfer to the mitochondrial matrix, leading to Ca^2+^ overload, opening of the permeability-transition pore, and further ROS generation [8], 51]. Additionally, the expression of the rough-ER tethering protein RRBP1 is suppressed, contributing to the failure of mitochondria to engage in beneficial interactions with ER sheets in obese hepatocytes [8].

Furthermore, altered mitochondrial output affects the body systemically. Impaired hepatic β-oxidation reduces ketone-body production during nutrient deprivation, while excess acetyl-CoA and citrate leaking into the cytosol drive *de novo* lipogenesis and steatosis [52]. In adipocytes, defective oxidative capacity limits thermogenesis and promotes lipid storage, while in pancreatic β-cells oxidative stress and mtDNA damage hinder glucose‐stimulated insulin secretion, worsening hyperglycemia [53]. These combined changes establish mitochondrial dysfunction as a central issue in the pathophysiology of obesity and a key target for therapeutic intervention.

## Mitochondria and adipose tissue

Diet-induced obesity (DIO) is characterized by an expansion of AT due to adipocyte hypertrophy accommodating increased triglyceride depots and altered cellular/mitochondrial metabolism. These changes have been associated with insulin resistance and, eventually, the development of type 2 diabetes (T2D) in some cases [54], 55].

In obesity, factors such as genetics, age, dietary habits, thermoneutrality and exposure to chemicals can lead to a transformation of brown AT (BAT) and beige AT, known as “whitening.” This transformation causes them to acquire characteristics of white AT (WAT) [56]. BAT whitening is sustained by changes in mitochondrial structure and function. A study conducted in different human and murine adipose depots, and mice lacking Apolipoprotein O (APOO), a protein in the mitochondrial cristae organizing system complex, has shown that APOO in adipocytes can worsen BAT whitening and DIO. Therefore, APOO could be a potential therapeutic target for obesity [57].

It has long been known that mitochondria are dysfunctional in obesity, as evidenced by decreases in ATP production, NADH dehydrogenase activity, mitochondrial membrane potential, and reduced expression of mitochondria-related genes like NRF-1, PGC-1α, COX1, and SOD in 3T3-L1 adipocyte cells. This dysfunction is attributed to AT hypoxia and the over-expression of inducible ATF3 in adipocytes [58].

A study using untargeted proteomics of mitochondria isolated from adipocytes, along with metabolite profiling of subcutaneous and intra-abdominal ATs in DIO vs. lean mice, was conducted to identify depot-specific alterations. The results indicate that, compared to lean mice, DIO mice demonstrate downregulation of protein components involved in the OXPHOS machinery within adipocytes, which may increase susceptibility to metabolic disease [59]. Another study involving young adult monozygotic Finnish twin pairs, both discordant and concordant for BMI, found that in obesity, subcutaneous abdominal adipocytes exhibit broad downregulation of oxidative pathways, mitochondrial transcripts, and OXPHOS protein levels, accompanied by increased expression of inflammatory pathways [60].

Colangeli et al. [61] hypothesized that gut dysbiosis, which is common in obesity, could affect AT metabolic activities by directly or indirectly affecting mitochondria in the AT. This may help explain why dietary modifications and anti-obesity drugs affect mitochondria in rat and mouse models of obesity as well as in humans [61], [62], [63], [64], [65].

Studies have shown that lipid-overloaded AT, a typical feature of obesity and obesity-related metabolic complications such as T2D and cardiovascular diseases, becomes dysfunctional by producing a wide array of secreted molecules. This fosters a pro-inflammatory microenvironment that can disrupt metabolic homeostasis across multiple organ systems [66]. Additionally, an emerging paradigm highlights the ability of adipocytes to extrude damaged mitochondria into the extracellular space, which are then transported via the bloodstream and taken up by recipient cells. This process is termed intercellular mitochondria transfer and could have significance in the context of obesity-associated AT dysfunction [67].

Poorly defined regulatory mechanisms connect adipocytes and immune cells in AT contributing to metabolic homeostasis [68]. In this context, macrophages acquire mitochondria from neighboring adipocytes *in vivo*, defining a transcriptionally distinct macrophage subpopulation. This indicates that intercellular mitochondria transfer between adipocytes and macrophages is a mechanism of immunometabolic crosstalk that, while regulating metabolic homeostasis physiologically, is impaired in cases of obesity [69].

In conclusion, mitochondrial structural and functional changes play a crucial role in the expanded, dysfunctional and “whitened” AT that often lead to insulin resistance. BAT “whitening” is strongly linked to mitochondrial changes and the loss of proteins such as APOO. Omic profiling suggests impaired oxidative pathways, and gut dysbiosis is associated with worsened mitochondrial function. Additionally, secreted inflammatory molecules and mitochondrial transfer also contribute to chronic metabolic derangements.

## Mitochondria and skeletal muscle

Although T2D is the prototypic metabolic disorder associated with mitochondrial dysfunction, impaired bioenergetic capacity of skeletal muscle mitochondria also occurs in obesity [70]. Ritov et al. have reported that individuals living with obesity or T2D exhibited two- to three-fold significantly reduced levels of the specific activity of NADH oxidase (per mg cardiolipin), NADH oxidase/citrate synthase, and NADH oxidase/beta-HAD ratios in their muscle biopsies compared to lean control subjects [71]. This bioenergetic imbalance is sustained by the accumulation of intramuscular lipids resulting in altered mitochondrial morphology, proteome, biogenesis, function, and turnover [72], 73].

Obesity and insulin resistance are connected to interactions between the sarcoplasmic reticulum and the dynamic tubular reticulum formed by mitochondria, collectively known as “mitochondrial networks.” This implies that metabolic disorders, through ion channels, can impact Ca^2+^ handling by these organelles [74]. Mitochondrial function can be influenced by changes in diet and physical exercise, with more noticeable structural and functional changes observed in subsarcolemmal (SS) mitochondria, which are located near the plasma membrane, compared to intermyofibrillar mitochondria (IMF) [72]. This subcellular distribution of mitochondrial dysfunction is similar to that found in individuals with T2D [71], where it may contribute to the development of muscle insulin resistance due to the significance of SS mitochondria in signal transduction and substrate transport. However, a conflicting study discovered an increase in proteins related to the tricarboxylic acid cycle ETC complex II, but a decrease in proteins forming ETC complexes I and III, as well as ATP synthase in subjects with obesity in IMF mitochondria, but not SS mitochondria [75].

The mitochondria-associated endoplasmic reticulum membrane (MAM) serves to connect mitochondria and the ER, allowing for the transfer of Ca^2+^ through the IP3R1-GRP75-VDAC1 complex. Excessive MAM formation can result in mitochondrial Ca^2+^ overload and dysfunction. PDK4 plays a role in stabilizing this complex at the MAM, and heightened PDK4 activity in obesity can lead to increased MAM formation and hindered insulin signaling. Inhibiting PDK4 can decrease MAM formation, enhance insulin signaling, and prevent associated mitochondrial and ER stress. *Pdk4* ^−/−^ mice demonstrate reduced susceptibility to insulin resistance. Conversely, artificially increasing MAMs can reverse these benefits [76].

Evidence from the obese mouse model suggests that an 8-week course of sucrose- and fat-restricted diet, combined with exercise, can improve metabolic indicators and muscle function through sucrose restriction, which reduces AMPK/AMPK and PGC-1α levels to that of control level [77].

In summary, mitochondrial dysfunction is not exclusive to T2D, but also affects obesity. It results in impaired bioenergetics in skeletal muscles and the storage of intramuscular lipids associated with alterations in mitochondrial morphology, proteomics and function. However, there is controversy surrounding the subcellular distribution of these changes. Cross-talks between the sarcoplasmic reticulum and mitochondria, which affects Ca^2+^ handling, as well as the excess formation of MAM membranes, contribute to insulin resistance in individuals with obesity. Making lifestyle changes can help improve metabolic health and muscle function.

## Mitochondria, oxidative stress, inflammageing and insulin resistance

Given that proper mitochondrial function is required for normal metabolism and health at multiple levels, the notion that a gradual decline of mitochondrial function occurs with age leading to progressive tissue damage via oxidative stress is one of the most intensively investigated factors contributing to aging [78]. Mutations in mitochondrial DNA (mtDNA) result in a variety of phenotypes, including premature aging and reduced lifespan. Additionally, mitochondrial dysfunction without somatic mutations is also a feature of normal aging across species from worms to humans [78]. Furthermore, mitochondrial dysfunction – with reduced expression of mtDNA and decreased levels of proteins involved in OXPHOS in muscle, liver and AT – occurs in many age-related diseases, including T2D and obesity [79]. Consistently, caloric restriction is associated with increased longevity through enhanced mitochondrial biogenesis and preserved function [78].

Inflammaging is a term used to describe a persistent, low-grade pro-inflammatory state that occurs with aging in mammals. This state is characterized by a reduction in autophagic efficiency, which compromises cellular maintenance mechanisms, leading to protein aggregation, the buildup of dysfunctional mitochondria, increased ROS production, oxidative stress, and the promotion of inflammaging through the activation of inflammasomes, particularly NLRP3 [80].

Under experimental conditions an elevated load of mitochondrial oxidants significantly contributes to insulin resistance by rapidly impairing insulin-regulated GLUT4 translocation [79]. Additionally, aging brings about a complete reshaping of immune-inflammatory responses, known as “immunosenescence,” which is mediated by altered mitochondrial metabolism through mitochondrial stress response with mitokines. Among these mitokines, GDF15, FGF21 and humanin increase with age and have anti-inflammatory effects as part of an adaptive immune-metabolic mechanism triggered by mitochondrial dysfunction to control inflammaging within a larger anti-inflammatory network [81].

Toll-like receptor 4 (TLR4) plays a crucial role in innate immunity. Experiments with genetically engineered mice have shown that disruption of TLR4 can reduce oxidative stress by reprogramming mitochondrial metabolism in visceral fat, thereby alleviating obesity-induced insulin resistance [82].

As individuals age, there is a general decrease in OXPHOS subunits. However, diabetic patients experience specific decreases in OXPHOS complexes alongside an increase in antioxidant response. Recent evidence suggests a connection between elevated thiol protein oxidation and reduced protein levels in AT mitochondria, particularly in individuals with T2D. This indicates potential issues with mitochondrial protein translocation and complex assembly. Additionally, the discovery that oxidized OXPHOS proteins are significantly reduced and changes in oxidized cysteine residues affect protein import via the redox-active mitochondrial intermembrane space assembly (MIA) pathway suggests that defects in mitochondrial protein translocation could be a key factor in mitochondrial dysfunction during both physiological aging and T2D [83].

The metabolic overload of mitochondria affects both muscle insulin resistance and β-cell dysfunction leading to incomplete β-oxidation, oxidative stress, accumulation of toxic lipid intermediates, and mitochondrial damage. These pathomechanisms may be counter-balanced by mitophagy and autophagy: mitophagy interrupts the cycle of oxidative stress and mitochondrial impairment, while autophagy enhances exercise-induced muscle glucose uptake, protects pancreatic β-cells from ER stress in diabetogenic conditions, and supports adipocyte differentiation in AT by facilitating mitochondrial clearance [84].

Sex hormones play a significant role in systemic and hepatic metabolic health [85], 86], and estrogen receptor beta (ERβ) is a potential mediator of this hormonally orchestrated metabolic homeostasis. González-Granillo et al. demonstrated that in non-ovariectomized female mice exposed to a high fat diet and selective pharmacological activation of ERβ with 4-(2-(3,5-dimethylisoxazol-4-yl)-1H-indol-3-yl)phenol, there is a tissue-specific effect [87]. This activation leads to enhanced mitochondria biogenesis in the liver and AT, resulting in improved fasting glucose levels, enhanced insulin sensitivity, and reduced liver steatosis, ultimately benefiting the whole metabolic response of the body to obesity [87].

In conclusion, inflammaging provides a crucial understanding of the connections between innate immunity, oxidative stress, autophagy, mitochondrial function, and insulin resistance that are characteristics of the natural aging process and exacerbated by diabetes and obesity. Managing inflammaging through lifestyle modifications and obesity treatment offers a promising approach to combating chronic non-communicable degenerative diseases.

## Mitochondria, diabetes and other cardio-nephro-metabolic diseases

First, we will describe general cell mechanisms. Then, we will examine the role of mitochondria in specific tissue types, including AT, pancreas, kidneys and the heart.

### General mechanism

#### Mitophagy

Mitophagy is a specialized form of autophagy regulated by either the PTEN-induced putative kinase 1 (PINK1)/Parkin-dependent pathway or receptors/adapters-dependent mitophagy. It is responsible for clearing dysfunctional mitochondria in both physiological and pathological conditions. Benign mitophagy refers to suppressed mitophagy that benefits mitochondrial homeostasis, while detrimental mitophagy describes excessive mitophagy that hinders the clearance of dysfunctional mitochondria [88].

#### Aberrant mitochondria

Remodeled mitochondria are pathogenically associated with obesity, insulin resistance, arterial hypertension, T2D, and metabolic syndrome. Therefore, inducing mitochondrial biogenesis along with improving the mitophagy machinery may prove helpful in clearing leaky, damaged aberrant mitochondria and preventing complications associated with the generation of ROS and the ROS-interactome [89].

### Specific cell types and tissues

#### Adipose tissue

**Figure 1:**
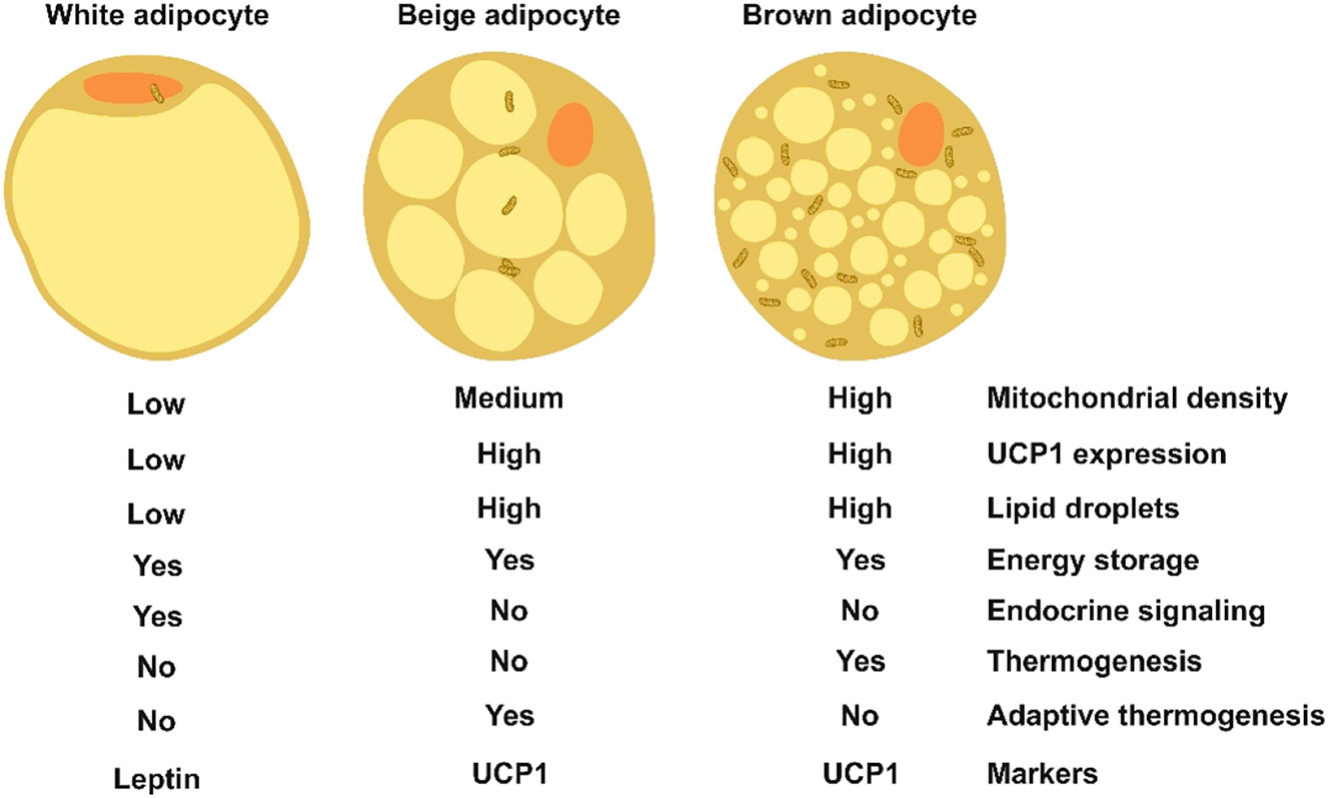
Similarities and differences among various adipocyte types. White, beige, and brown adipocytes have certain similar and different features. Illustration adapted from [55] under the terms and conditions of the creative commons attribution (CC BY) license (https://creativecommons.org/licenses/by/4.0/). UCP1- uncoupling protein 1.

AT plays a crucial role in energy homeostasis by demonstrating significant metabolic flexibility. However, obesity and diabetes often compromise this flexibility due to mitochondrial dysfunction within adipocytes. This dysfunction leads to inefficient lipid handling and, when combined with increased oxidative stress, sustains the systemic metabolic disruptions central to obesity and its complications [55]. Therefore, examining the distinct morpho-functional characteristics of the three types of adipocytes, white, beige, and brown is essential in understanding the fundamental role of AT in obesity. Figure 1, adapted from [55], schematically illustrates the structure, functions, and biomarkers of these AT cell types.

In short, obesity is associated with white adipocytes that significantly impair lipid metabolism and exacerbate oxidative stress, thereby contributing to metabolic dysfunction. Mitochondrial impairment in these cells diminishes thermogenic potential, limiting optimal energy expenditure in brown adipocytes. Additionally, beige adipocytes possess both morphological and functional characteristics of white and brown adipocytes. While they resemble white adipocytes, they retain the ability to convert into mitochondria-rich, energy-consuming cells when exposed to metabolic stressors such as cold exposure or fasting [55]. Therefore, the mitochondrial dynamics specific to each cell type contribute to variable adipocyte metabolic phenotypes through specialized networks comprising transcription factors, co-activators, and enzymes that orchestrate energy homeostasis at the cellular level.

#### Pancreatic β-cells

Dysfunctional pancreatic β-cells are a prerequisite for the development of glucose-stimulated insulin secretion (GSIS) and T2D. Therefore, it is important to characterize the subcellular mechanisms that precede GSIS. Addressing this research question, Dos Reis Araujo et al. have elegantly shown that obesity increases ER-mitochondria contact points [90]. These changes in mitochondrial-associated membranes precede GSIS impairment, indicating that these interactions could be the ideal target to prevent further disruption in β-cell function.

#### Kidneys

Two studies have contributed to advancing our understanding of the role of mitochondria in obesity-associated chronic kidney disease (CKD). Munusamy et al. compared two normotensive genetic mouse models of obesity, leptin-deficient ob/ob mice and hyperleptinemic melanocortin-4 receptor-deficient mice (LoxTB *Mc4r* ^−/−^), with their respective lean controls [91]. These authors found that alterations to mitochondria, which involve reduced cellular ATP levels may be implicated in obesity-induced renal injury. The type and severity of mitochondrial and ER dysfunction depend on whether leptin is present or absent.

Szeto et al. found that significant changes occur in mitochondrial structure in glomerular endothelial cells, podocytes, and proximal tubular epithelial cells after 28 weeks of a high-fat diet in C57BL/6 mice [92]. However, treatment with SS-31, a tetrapeptide that targets cardiolipin and protects mitochondrial cristae structure without affecting body weight, insulin resistance or glycemia, during high-fat diet preserved normal mitochondrial structure in all kidney cells. It also restored renal AMP kinase activity, prevented intracellular lipid accumulation, endoplasmic reticulum stress, and apoptosis, as well as the loss of glomerular endothelial cells and podocytes, mesangial expansion, glomerulosclerosis, macrophage infiltration, and the upregulation of proinflammatory (TNF-a, MCP-1, NF-κB) and profibrotic (TGF-β) cytokines. This study suggests that agents involved in mitochondria protection can overcome renal lipotoxicity representing an upstream target for therapeutic innovation in this field.

#### Heart

It is widely accepted that obesity among younger individuals leads to premature cardiac aging, ultimately increasing the risks of heart failure. Niemann et al. conducted a study analyzing right atrial cardiomyocytes for mitochondrial function, markers of apoptosis, cardiac load or metabolism, and oxidative stress parameters in 60 men who underwent cardiac surgery, stratified by BMI, metabolic status, and age [93]. The data showed that obesity disrupts mitochondrial biogenesis and function in cardiomyocytes regardless of age, resulting in increased oxidative stress and up to 30 % telomere shortening, higher markers of cardiac load and apoptosis, as well as altered glucose metabolism and adipocytokine levels. However, among those over 70, obesity only caused minor additional changes compared to their normal-weight peers.

In an experiment conducted by Martinez-Abundis et al., mitochondrial transition pore opening was analyzed after exposing neonatal rat ventricular myocytes to leptin for 24 h. The data showed that leptin can enhance calcium-mediated, Stat3-dependent pro-apoptotic effects by increasing mitochondrial transition pore opening, regardless of its hypertrophic actions [94]. This study suggests that leptin plays a role in mitochondrial dysfunction and apoptosis in the diseased myocardium, particularly when excessive intracellular calcium accumulates.

All these findings demonstrate that mitochondrial structural and functional integrity plays a crucial role in regulating cellular energy metabolism and influencing the vulnerability of various target organs to functional impairment associated with obesity. This is a common feature in various clinically heterogeneous conditions including visceral obesity, T2D, CKD, and heart failure. Strategies focused on improving mitochondrial health, such as promoting biogenesis, enhancing structural stability, and preventing dysfunction caused by metabolic stress and lipotoxicity, show promise in reducing the progression of metabolic disorders and maintaining the function of AT, pancreatic β-cells, kidneys, and the heart in individuals with obesity.

## Mitochondria and steatotic liver disease

States of obesity are variably associated with chronic liver disease (CLD), due to alcohol-related and viral etiologies. However, the prototypic manifestation of obesity-associated CLD is metabolic dysfunction-associated steatotic liver disease (MASLD), and obesity is a major risk factor for progression to MASLD-cirrhosis and MASLD-hepatocellular carcinoma (HCC) through lipotoxic, proinflammatory, pro-fibrotic, and carcinogenic pathomechanisms [95]. During alcohol-induced hepatotoxicity assessed in hepatoma cell line VL-17A mitochondria adapt in response to ethanol with the development of ethanol-induced hepatic mitochondrial changes mediated through dynamin-1-like protein (Drp1) [96]. Therefore, hepatic megamitochondria are a hallmark of alcoholic liver diseases [96]. However, in their pioneering paper published in 1999, Caldwell et al. observed megamitochondria exhibiting organelle swelling and containing linear crystalline inclusions in eight out of 10 NASH patients [97]. Subsequent investigation has shown that gene knockout of OPA1, a mitochondrial dynamin-related GTPase that mediates mitochondrial fusion, prevents megamitochondria formation and liver damage in a mouse model of NASH induced by a methionine-choline-deficient (MCD) diet [98]. Consistently, the utilization of anti-sense oligonucleotides against OPA1 at the disease onset effectively prevented megamitochondria formation and liver pathologies in the MCD model and even when applied after the development of overt NASH targeting OPA1 effectively regressed megamitochondria, and reduced steatosis, inflammation, and fibrosis [99].

Obesity causes significant reorganization of MAMs in the liver, resulting in mitochondrial calcium overload, reduced mitochondrial oxidative capacity, and increased oxidative stress. Experimental studies have shown that enhancing ER-mitochondria interactions can lead to oxidative stress and disrupt metabolic homeostasis. Conversely, reducing the levels of PACS-2 or IP3R1, proteins essential for ER-mitochondria tethering and calcium transport, respectively, has been found to enhance mitochondrial oxidative function and improve glucose metabolism in animal models of obesity [100].

To maintain their functionality, mitochondria undergo dynamic shape changes through fusion and fission [101]. When damaged, mitochondria can enlarge into megamitochondria, organelles characterized by larger size, pale matrix, and marginal cristae, often observed in diseases affecting energy-demanding cells such as hepatocytes and cardiomyocytes [102]. While megamitochondria may aid cells in surviving temporary environmental stress, prolonged formation can exacerbate metabolic disorders and disease progression [102].

Greatorex et al. found that NOX4 and NFE2L2 levels increase early in liver disease to help prevent NASH and fibrosis [103]. Mitochondria-derived ROS activate NFE2L2, triggering NOX4 expression and further H_2_O_2_ production, which amplifies antioxidant defenses. In hepatocytes, NOX4 deletion or inhibition reduces ROS but weakens antioxidant responses, leading to more mitochondrial damage, impaired insulin signaling, and increased cell death under oxidative stress. In high-fat diet-fed mice, loss of hepatocyte NOX4 promotes liver damage, inflammation, and NASH progression, while NOX4 overexpression protects from NASH and fibrosis. Collectively, data suggest that, in obesity, mitochondria- and NOX4-derived ROS act in concert to drive a NFE2L2 antioxidant defense response to attenuate oxidative liver damage and progression to fibrosing NASH [103].

Mitochondria-associated microRNAs (miRNAs) have recently been identified as important regulators of cellular metabolism, with the capability to influence mitochondrial fusion and fission processes [104]. The study by Ji et al. investigating the involvement of mitochondria-related miRNAs in obesity has demonstrated that miR-141-3p contributes to mitochondrial dysfunction by inhibiting PTEN in mice subjected to a high-fat diet [105]. The results indicate that elevated hepatic miR-141-3p expression is associated with impaired mitochondrial function, leading to reduced ATP production and increased oxidative stress. These findings underscore the importance of mitochondrial regulation in the development of metabolic disturbances associated with obesity.

Diet and sex strongly influence the risk of MASLD and metabolic dysfunction-associated steatohepatitis (MASH) [106], 107]. Mitochondrial pathobiology may play a role in mediating these risk factors. Barrios-Maya et al. have shown that palmitic acid and palmitoyl-CoA are directly involved in MASLD aggravation through pathomechanisms involving cyt-c release and membrane permeability transition, a key process of apoptosis [108].

Female mice fed a cafeteria diet develop more severe accumulation of liver steatosis and oxidative stress than male controls because of unbuffered ER stress. Moras Mewes et al. have shown that increased mitochondrial ROS generation associated with a decrease in the antioxidant defense capacity, contributes to the development of ER stress in the livers of female mice fed a cafeteria diet [109].

Increasing evidence supports the notion that damage and dysfunction of mtDNA play significant roles in the development of certain conditions related to mitochondria, particularly diabesity. Zhang et al., using a genetically engineered transgenic mouse model expressing Human 8-oxoguanine-DNA glycosylase 1 (hOGG1), have shown that mitochondrial hOGG1 overexpression led to more free radicals and major mtDNA deletions [110]. In hOGG1 TG mice, obesity was driven by increased fat from overeating, linked to upregulated orexigenic and lipogenic genes and downregulated anorexigenic, thermogenesis, and fatty acid oxidation genes. These mice also showed diffuse liver steatosis, female infertility, and higher rates of malignant lymphoma. Collectively, these findings demonstrate the notion that enhanced oxidative DNA damage processing may be an important factor in human metabolic syndrome, infertility, and malignancy [110].

Given the close relationship between MASLD and CKD [111], it is important to note that megamitochondria are commonly observed in podocytes during diabetic nephropathy and that insufficient autophagy flux may underlie this effect [112]. The finding that mitochondria-targeted sulfide delivery molecules could potentially provide a novel therapeutic approach to the treatment or prevention of obesity-related metabolic disorders [113].

**Figure 2:**
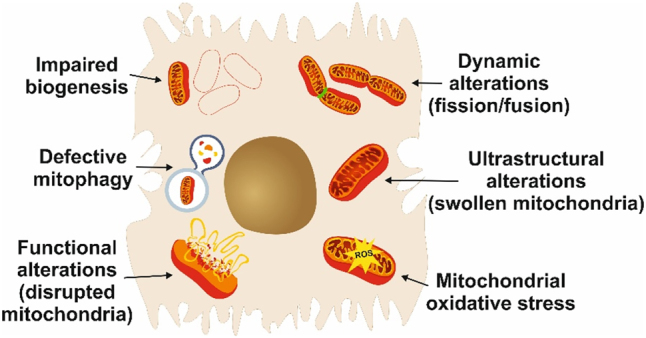
Mitochondrial abnormalities in MASLD. Schematic illustration of the wide spectrum of mitochondrial changes observed in hepatocytes during MASLD. Modified from [114] under the terms and conditions of the creative commons attribution (CC BY) license (https://creativecommons.org/licenses/by/4.0/).

In conclusion excessive ER-mitochondrial coupling serves as an essential component of organelle dysfunction in obesity that may contribute to the development of metabolic pathologies such as insulin resistance and diabetes. Morphologically aberrant mitochondria, for example, megamitochondria with crystalline inclusions, are common in MASH, alcohol-related liver disease, and diabetic nephropathy, indicating that reversal of mitochondrial dysfunction owing to mitochondria-directed innovative therapeutic approaches might potentially benefit clinically heterogeneous though pathogenically related pathologies. Figure 2 summarizes the spectrum of mitochondrial abnormalities observed in MASLD.

## Mitochondria and female reproductive health

Reproductive health and metabolic function are closely related, with mitochondria playing a key role in this connection [107], 115]. In the following, we explore the evidence suggesting that oocytes and the placenta are involved in modulating reproductive health outcomes in obese women.

### Oocytes

Since mitochondria are inherited maternally, abnormalities in these organelles may constitute a pathway for the transmission of metabolic dysfunction [116]. Maternal DIO at birth can lead to increased mitochondrial density and potential dysfunction in offspring’s primordial oocytes, which may be a marker for heightened bioenergetic capacity or early damage. At weaning, the impact on oocyte mitochondria is less clear but could be influenced by an offspring’s own diet. This implies that maternal obesity could program an offspring for future metabolic issues and highlights how crucial the pre-gestational and early-life period is for female reproductive health, potentially impacting future generations. Within this context, Marei et al. found that the oocyte mitochondrial structural abnormalities previously reported in adult offspring from DIO mothers were not detected in the primordial follicle oocytes at birth and were only detected at weaning [117]. Zhang et al. further explored the molecular mechanisms involved in associating obesity with oocyte abnormalities [118]. The study demonstrates that Inverted-formin two plays a key role in the maturation of both mouse and porcine oocytes by regulating actin polymerization and tubulin acetylation, which are essential for mitochondrial function. Furthermore, deficiency of Inverted-formin 2 may contribute to obesity-induced oocyte defects.

Saben et al. after administering a high-fat/high-sugar diet to female mice from the preconception period through weaning, subsequently observed outcomes in three successive generations of offspring, all maintained on a control diet [119]. The results demonstrated that female progeny of obese mothers exhibited impaired peripheral insulin signaling, which was correlated with mitochondrial dysfunction, as well as alterations in mitochondrial dynamics and complex proteins within skeletal muscle. Notably, this mitochondrial phenotype persisted across the female germline and was transmitted to the second and third generations.

The findings suggest that maternal programming of metabolic disease may be heritable through the female germline, and that the transfer of aberrant oocyte mitochondria to subsequent generations could contribute to an elevated risk for insulin resistance.

Boudoures et al. examined the impact of inactive mitophagy in oocytes on the maternal transmission of dysfunctional mitochondria [120]. The findings demonstrated that mitophagy in oocytes functions distinctly from that in somatic cells, and that an absence of mitophagy under high-fat/high-sugar conditions facilitates the transfer of dysfunctional mitochondria from the oocyte to the blastocyst.

### Placenta

Maternal obesity can negatively impact pregnancy outcomes by affecting placental health. A lipotoxic placental environment is linked to higher levels of inflammation and oxidative stress, which can disrupt mitochondrial function. This disruption can lead to placental dysfunction and poor pregnancy outcomes. A study by Mandò et al. by examined mitochondrial DNA and electron microscopy morphology in the placentas of 47 singleton pregnancies during elective cesarean sections. They discovered that mitochondrial alterations, along with changes in placental energetics, are common in pregnancies of obese women [121]. These findings may help explain why individuals with obesity often experience adverse pregnancy outcomes.

### Therapeutic implication

Previous studies have shown that dapagliflozin (DAPA) improves body composition and metabolic disorders in obese women. A study by Liu et al., tested whether DAPA could ameliorate high-fat diet (HFD)-induced ovarian dysfunction in female mice and through which molecular mechanisms [122]. Data have shown that DAPA attenuates glucose accumulation and enhances mitochondrial function in granulosa cells under high-fat conditions, potentially through suppression of SGLT2 expression, ultimately reducing pyroptosis and improving follicular development in obesity.

Therefore, obesity impairs reproductive outcomes in women and has the potential to pass on the risks of insulin resistance to future generations. Both oocyte and placental mitochondria play a role in the impaired pregnancy outcomes of women with obesity, as well as in transmitting metabolic dysfunction to future generations. Drug treatment has shown promise in improving oocyte dysfunction, but further studies are required to determine the risk/benefit ratio in this area. Additionally, more research is needed on the role of mitochondria in male infertility in obese men.

## Mitochondria and cancer

Obesity is a risk factor associated with several types of cancer [123], including (postmenopausal) female breast, colorectal, uterine, pancreatic, esophageal, gallbladder, liver, kidney, and thyroid cancers [124], [125], [126]. Otto Warburg observed that cancer cells have a unique characteristic of absorbing and fermenting glucose to lactate, even in the presence of oxygen, a process known as aerobic glycolysis. He proposed that defects in mitochondrial respiration are the fundamental cause of both aerobic glycolysis and cancer [127], 128]. This traditional perspective indicating glycolysis as the primary metabolic pathway for energy generation and anabolic growth in cancer cells, has led to the development of advanced imaging technologies still widely used in clinical practice. The “Warburg effect” is the basis for tumor imaging by fluorodeoxyglucose-position emission tomography (FDG-PET), which is commonly used [129]. However, recent evidence shows that mitochondria play a crucial role in all stages of oncogenesis, not only om their bioenergetic functions, but also as contributors of precursors for tumor growth, regulation of redox balance and calcium homeostasis, participation in transcriptional control, interaction with the host immune system, and mediators of cell death processes [130]. Therefore, mitochondria are promising targets for novel therapeutic opportunities aimed at mitochondrial metabolism in cancer treatment [131].

A recent *in vitro* study used a 3D culture model to investigate the impact of an ELIT cocktail (17β-estradiol, leptin, IL-6, and TNFα), which mimics obesity-related inflammation in postmenopausal women [132]. The data suggest that antioxidants, insulin-sensitizers, and identified mitochondrial markers could be valuable tools for prognostication and the development of more effective therapies for individuals with obesity-related luminal breast cancer.

## Mitochondria, diets, exercise, and drugs

**Figure 3:**
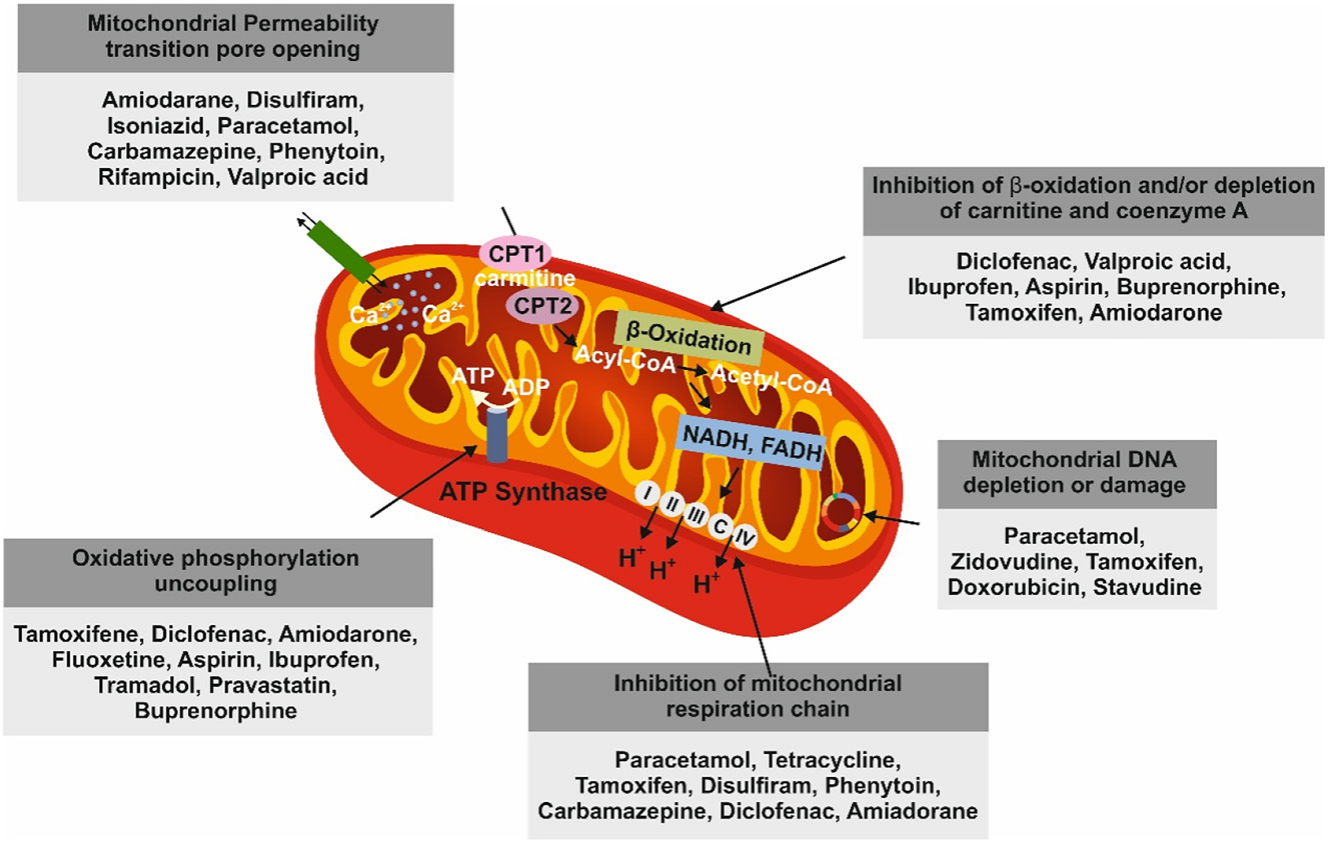
Drug-induced mitochondrial toxicity. Schematic illustration of the specific mitochondrial sites (i.e. permeability pore, respiratory chain, phosphorylation uncoupling, beta-oxidation and DNA) where individual drug classes can interfere with mitochondrial function. Reproduced with modification from [137] under the terms of the creative commons attribution license (CC BY).

Nutrients, mitochondrial function, and apoptosis are closely interconnected, with important implications for aging and metabolic disorders. Evolutionarily, the availability of nutrients may have shaped divergent mitochondrial roles: facilitating energy production via OXPHOS to support cellular function and mediating the release of apoptotic proteins to initiate programmed cell death [133]. With this evolutionary backdrop, diets, exercise, and drugs significantly affect mitochondria by influencing their number (biogenesis), quality control (mitophagy), shape (dynamics), and energy production efficiency (Figure 3). Dietary habits play a crucial role in mitochondrial function [134] and exercise is one of the most effective ways to improve mitochondrial health [135]. Drugs have a variable effect on mitochondria, with some drugs improving function in specific contexts while many others such as statins, anti-diabetics, anti-epileptics, nonsteroidal anti-inflammatory drugs (NSAIDs), anti-depressants, and certain antibiotics carry a risk of toxicity [136], 137]. Figure 3 illustrates the mechanisms through which individual drug classes interfere with mitochondrial vital activities [137].

### Diets

Dietary fatty acids play a significant role in mitochondrial function, by modifying the architecture of mitochondrial membranes and influencing metabolic pathways directly. The effects of different types of fatty acid are distinct: omega-3 polyunsaturated fatty acids, like docosahexaenoic acid (DHA) and eicosapentaenoic acid (EPA), are linked to improved mitochondrial performance, while diets high in saturated fatty acids have been associated with compromised mitochondrial function and increased production of ROS [138].

Polysaccharides are a prominent class of bioactive compounds found in natural products, with great potential in managing metabolic disorders. Recent research has highlighted the promising role of natural polysaccharides in addressing obesity, a condition in which mitochondria play a key role in initiation, progression, and regulation [139]. Natural polysaccharides have also shown effectiveness in improving mitochondrial dysfunction through various mechanisms. The anti-obesity properties of these compounds are closely linked to their structural attributes, such as molecular weight, monosaccharide composition, glycosidic linkages, conformational characteristics, extraction methods, and their ability to enhance mitochondrial oxidative respiration, reduce oxidative stress, and maintain mitochondrial mass homeostasis [139].

### Exercise

De Almeida et al., using transmission electron microscopy, found that 1 h of acute exercise increases the length of contact between lipid droplets and mitochondria within skeletal muscle, regardless of obesity or T2D. Contact length was correlated with the rate of fat oxidation during exercise [140]. This study suggests that exercise facilitates contact, and therefore potential functional collaboration, between lipid droplets and the mitochondrial network irrespective of metabolic comorbidity. Heo et al. studied 4-week-old C57BL/6 mice with DIO and found that treadmill exercise improved skeletal muscle structure and reduced apoptosis in obese skeletal muscles [141]. These studies clearly indicate that the physiology of mitochondria in skeletal muscle is implicated in the beneficial metabolic effects of exercise.

### Associations of diets and exercise

Obesity is associated with mitochondrial impairment, while exercise training and mitochondrial nutrients promote mitochondrial function in skeletal muscle [142]. Lee and colleagues found that using 8 weeks of treadmill exercise and sucrose or fat restriction diets in obese mice showed that exercise or a sucrose and fat restriction diet improved metabolic indicators and muscle function [77]. Specifically, exercise increased the protein expression level of PGC-1α in obesity, while sucrose restriction reduced the phosphorylated AMP-activated protein kinase (pAMPK)/AMPK ratio and PGC-1α to the control level.

### Drugs and mitochondria

Aberrant production of ceramides, molecules whose side chain length determines lipotoxicity (those composed of C16:0 or C18:0 side chains are toxic whereas those with C24:0 or C24:1 are not) is a hallmark of obesity and strongly linked to metabolic dysfunction [143]. Counteracting the deleterious effects of diets rich in saturated fat either by inhibiting synthesis or by promoting degradation of ceramides mitigates insulin resistance and ectopic lipid accumulation. However, drugs that safely and selectively target ceramide metabolism have failed to translate into metabolic benefit in human trials so far [143]. Despite this setback, Jayashankar et al. have demonstrated that the synthetic sphingolipid SH-BC-893 exerts notable beneficial effects, including inhibition of palmitate- and ceramide-induced mitochondrial fission, preservation of mitochondrial function, and prevention of ER stress *in vitro* [144]. Similar improvements were observed in tissues from HFD-fed mice. Within 4 h of oral administration, SH-BC-893 normalized mitochondrial morphology in the liver and brain of HFD-fed mice, enhanced mitochondrial function in WAT, and corrected abnormal plasma leptin and adiponectin levels. Collectively, these results suggest that the sphingolipid analog SH-BC-893 effectively and rapidly mitigates ceramide-induced mitochondrial dysfunction, thereby addressing DIO and its associated metabolic complications.

Liu et al. examined the effects of sesamol, a potent natural antioxidant and anti-inflammatory phenolic compound derived from sesame oil, on adiposity, metabolic disturbances, and mitochondrial-lipid metabolism in 3-month-old C57BL/6J mice fed a Western diet [145]. The findings demonstrate that sesamol attenuated body weight gain, improved diet-induced insulin resistance, partially normalized serum and hepatic lipid profiles, and suppressed diet-induced hepatic lipogenesis by modulating mitochondria-related genes involved in triglyceride and cholesterol metabolism. Notably, sesamol reduced both the mass and adipocyte size of WAT and BAT through upregulation of mitochondrial genes such as Pgc1a and Ucp1. Additionally, sesamol inhibited lipid accumulation induced by differentiation and exposure to mitochondrial metabolic inhibitors (oligomycin and antimycin A) in 3T3-L1 adipocytes. This study provides compelling evidence that sesamol supplementation reduced adipocyte size and adipogenesis in DIO by regulating mitochondria lipid metabolism.

Dodecyltriphenylphosphonium (C12TPP), a membrane-permeable cation, promotes the recycling of fatty acids within artificial lipid membranes and mitochondria. C12TPP can dissipate the mitochondrial membrane potential and may influence overall energy expenditure as well as body weight in both animal models and humans [146]. In this context, Kalinovich et al. examined the metabolic effects of C12TPP using isolated brown-fat mitochondria, cultured brown adipocytes, and an *in vivo* mouse model. The data suggest that C12TPP mitigates DIO by decreasing food intake, elevating resting metabolic rate, and promoting fatty acid oxidation [146].

The pro-inflammatory activation of AT macrophages (ATMs) has been causally implicated in obesity and related disorders. Extensive research has demonstrated the critical role of mitochondrial metabolism in macrophage activation. However, there remains a significant lack of pharmacological agents that specifically target the mitochondrial metabolism of ATMs for the treatment of obesity-associated diseases. Wang et al. investigated the properties of a near-infrared fluorophore (IR-61), which preferentially accumulates in the mitochondria of ATMs and demonstrates therapeutic effects on DIO, insulin resistance associated with obesity, and steatotic liver disease [147]. IR-61 suppresses classical activation of ATMs by elevating mitochondrial complex levels and promoting OXPHOS through the ROS/Akt/Acly signaling pathway. Collectively, these findings suggest that targeted enhancement of OXPHOS in ATMs can alleviate chronic inflammation and obesity-related disorders, indicating that IR-61 may serve as an anti-inflammatory agent with potential for treating obesity-associated conditions by specifically targeting ATM mitochondria. Moreover, research has evaluated the positive impacts of phytopharmaceuticals on mitochondrial function [148], 149]. However, the translational potential of these findings is yet to be established.

In sum, diet, physical activity, and pharmacological agents significantly impact on mitochondrial function by regulating biogenesis, mitophagy, dynamics, and overall energy production efficiency. Dietary patterns play a crucial role in controlling mitochondrial activity. Regular exercise is widely recognized as one of the most effective strategies for improving mitochondrial health. Medications exert varying effects on mitochondria, with some drugs potentially enhancing mitochondrial function in certain situations, while many others may pose a risk of mitochondrial toxicity. Additionally, recent research suggests that specific natural compounds and targeted small molecules can influence mitochondrial pathways to combat obesity and metabolic disorders. These findings highlight the potential for lifestyle changes and pharmacological interventions to work together in supporting mitochondrial health and preserving metabolic functions.

## Limitations

This scoping review has several limitations that should be considered when interpreting its findings. First, the literature search was intentionally restrictive, relying on PubMed and Web of Science and initially using only the Title keywords “Obesity” AND “Mitochondria,” with subsequent expansion through examination of cross-references. Therefore, relevant studies indexed in other databases or not using these terms in the title may have been missed. Second, we limited inclusion to English-language publications, which may introduce language bias. However, this limitation is shared by the vast majority of published reviews. Third, the marked heterogeneity of study designs, populations, mitochondrial endpoints, and metabolic outcomes precluded quantitative synthesis. For this reason we were unable to derive pooled effect estimates or formally compare intervention effects across studies. Fourth, many of the included sources were preclinical animal or *in vitro* studies with variable reporting quality and limited external validity. Our decision not to undertake a formal risk-of-bias or quality assessment at the study level, is consistent with scoping review methodology but constrains causal inference. Finally, our search was current up to 10 November 2025, and more recent evidence could not be captured.

## Conclusion and research agenda

The role of mitochondria, which has been discussed in previous chapters, is summarized in Table 1.

**Table 1:** Overview of mitochondria in obesity medicine.

Highlights	Findings	Refs.
Physiological functions	Mitochondria are double-membrane organelles that produce energy through oxidative phosphorylation at the inner membrane. Their matrix hosts the TCA cycle, fatty acid β-oxidation, amino acid breakdown, and the urea cycle, coordinating major metabolic pathways. They also synthesize haem, Fe–S clusters, cardiolipin, and steroid hormones. While low mitochondrial ROS levels signal cellular processes, high levels cause damage and activate mitophagy	[18], [19], [20], [21], [22], [23], [24], [25], [26], [27], [28], [29], [30], [31], [32], [33]
	Mitochondria form networks through fusion and fission, aiding stress management and energy balance. In hepatocytes, mitochondria connect with rough ER in high fatty acid oxidation zones and with smooth ER for calcium signaling and lipid transfer. These interactions support key mitochondrial roles in cell signaling, energy production, and gene regulation, with fasting and liver zone differences mainly affecting mitochondria-ER contacts in select areas	[34], [35], [36], [37], [38], [39], [40], [41], [42]
Pathological associations	**Adipose tissue** – Obesity and diabetes reduce metabolic flexibility in AT by impairing mitochondria, leading to poor fat processing and increased oxidative stress. White adipocytes worsen these dysfunctions, while malfunctioning brown adipocytes lose heat production; beige adipocytes can adapt by increasing energy burning under stress	[55]
	**Pancreatic β-cells** – Impaired pancreatic β-cells are essential for developing GSIS and T2D. Obesity raises ER-mitochondria contact points, which occur before GSIS impairment. These interactions may be promising targets to prevent further β-cell dysfunction	[90]
	**Kidneys** – Experimental studies indicate that changes in mitochondria and reduced ATP may contribute to obesity-related kidney injury, influenced by leptin status. Mitochondria-protective agents may help prevent renal lipotoxicity.	[91], 92]
	**Heart** – Preserved mitochondrial structure and function are key to cellular energy metabolism and affect how organs respond to obesity-related impairment, common in conditions like visceral obesity, T2D, CKD, and heart failure	[93], 94]
	**Liver** – Obesity accelerates ALD and MASLD progression to cirrhosis and HCC due to lipotoxicity, inflammation, and fibrosis. Megamitochondria signal ALD and NASH; early OPA1 inhibition can prevent them in animals. Short-term mitochondrial enlargement may protect, but persistent megamitochondria worsen liver disease. In obesity, mitochondrial and NOX4-driven ROS trigger antioxidants that help limit liver damage. Mitochondrial biology also impacts how diet and sex affect MASLD/MASH risk	[95], [96], [97, 99], 100], 102], 103], 106], 107]
	**Female reproductive health** – Reproductive health and metabolism are closely linked through mitochondrial function. Maternal transmission of abnormal oocyte mitochondria may increase insulin resistance risk in offspring. Additionally, maternal obesity can impair placental health, leading to inflammation, oxidative stress, and mitochondrial dysfunction, negatively affecting pregnancy outcomes	[107], 115]
	**Cancer** – Obesity increases the risk of several cancers, such as postmenopausal breast, colorectal, uterine, pancreatic, esophageal, gallbladder, liver, kidney, and thyroid cancers. The “Warburg effect” underlies tumor imaging with FDG-PET, and recent studies highlight mitochondria’s critical role in all stages of cancer development	[123], [124], [125], [126, 129]
Therapeutic strategies	Diet, physical activity, and pharmacological agents exert significant effects on mitochondria by modulating biogenesis, mitophagy, mitochondrial dynamics, and the efficiency of energy production. Dietary patterns significantly influence mitochondrial function. Engaging in regular physical activity is among the most effective approaches for enhancing mitochondrial health. Drugs exhibit diverse effects on mitochondrial function; while some may enhance activity under certain conditions, numerous agents, including statins, antidiabetic medications, antiepileptics, NSAIDs, antidepressants, and specific antibiotics, pose a potential risk of mitochondrial toxicity	[134], [135], [136], [137]

ALD, alcohol-related liver disease; AT, adipose tissue; ATP, adenosine triphosphate; CKD, chronic kidney disease; ER, endoplasmic reticulum; FDG-PET, ^18^F-fluorodeoxyglucose positron emission tomography; Fe-S, iron-sulfur; GSIS, glucose-stimulated insulin secretion; HCC, hepatocellular carcinoma; MASLD, metabolic dysfunction-associated steatotic liver disease; MASH, metabolic dysfunction-associated steatohepatitis; NASH, non-alcoholic steatohepatitis; NOX4, NADPH, oxidase 4; NSAIDs, non-steroidal anti-inflammatory drugs; OPA1, optic atrophy protein 1; ROS, reactive oxygen species; T2D, type 2 diabetes; TCA, tricarboxylic acid (cycle).

It is important to recognize that the mechanisms, cell types, and tissues described operate cooperatively within systemic metabolic health, with mitochondria serving as fundamental regulators. Correspondingly, mitochondrial dysfunction can contribute to pathophysiological changes in various target organs, such as the endocrine pancreas, liver, kidney, and heart.

Examining the association between obesity, T2D, and mitochondrial function provides valuable insight into the underlying pathophysiology of these conditions [150]. Mitochondria, as essential organelles, facilitate cellular energy production through OXPHOS. Obesity has been linked to mitochondrial dysfunction, leading to impaired biogenesis, diminished oxidative capacity, and increased oxidative stress. These dysfunctions result in inefficient energy utilization, which may underlie the multiple metabolic derangements commonly associated with obesity. Furthermore, compromised mitochondrial function adversely impacts insulin signaling pathways. Reduced oxidative capacity elevates ROS, triggers stress-related signaling cascades, and disrupts insulin activity, ultimately promoting insulin resistance in peripheral tissues [150].

The conceptualization of mitochondria has evolved significantly in recent years. Beyond being regarded merely as the cell’s powerhouse, mitochondria are now recognized for their critical involvement in diverse biological functions and their emerging role as a signaling platform. This shift has spurred renewed interest in investigating potential links among obesity, mitochondria, cellular signaling, apoptosis, impaired systemic metabolic health, and aging [89], 151]. Future research should focus on identifying non-invasive biomarkers indicative of mitochondrial health and function, as well as exploring the efficacy of lifestyle interventions and pharmacological treatments in this domain.

## Supplementary Material

Supplementary Material

Supplementary Material
