# Supplementary material for: Mitochondrial medicine in obesity: a scoping review
Source: Open Med (Wars). 2026 Apr 8;21(1):20261407. doi: 10.1515/med-2026-1407 (PMC13068878; doi:10.1515/med-2026-1407)
Supplement: Supplementary file 2 — Supplementary Material [file j_med-2026-1407_suppl_002.docx]

Supplementary References (List of excluded articles)

1. Kozak LP, Koza RA. Mitochondria uncoupling proteins and obesity: molecular and genetic aspects of UCP1. Int J Obes Relat Metab Disord. 1999;23 Suppl 6:S33-7. doi: 10.1038/sj.ijo.0800941

2. Côté HC, Brumme ZL, Chan JW, Guillemi S, Montaner JS, Harrigan PR. HIV therapy, hepatitis C virus infection, antibiotics and obesity, a mitochondria killer mix? AIDS 2006;20(9):1343-5. doi: 10.1097/01.aids.0000232250.89404.00

3. Abel ED. Obesity stresses cardiac mitochondria even when you are young. J Am Coll Cardiol. 2011;57(5):586-9. doi: 10.1016/j.jacc.2010.09.039

4. Ocloo A, Dongdem JT. Mitochondria as pharmacological targets: the discovery of novel anti-obesity mitochondrial uncouplers from Africa's medicinal plants. Afr J Tradit Complement Altern Med. 2011;9(2):256-9. doi: 10.4314/ajtcam.v9i2.11

5. Nichols TW Jr. Mitochondria of mice and men: moderate magnetic fields in obesity and fatty liver. Med Hypotheses 2012;79(3):287-93. doi: 10.1016/j.mehy.2012.05.006

6. Aguilar A. Obesity: Shielding mitochondria from lipotoxicity prevents renal injury. Nat Rev Nephrol. 2016;12(10):580. doi: 10.1038/nrneph.2016.132

7. Song W, Owusu-Ansah E, Hu Y, Cheng D, Ni X, Zirin J, et al. Activin signaling mediates muscle-to-adipose communication in a mitochondria dysfunction-associated obesity model. Proc Natl Acad Sci U S A 2017;114(32):8596-8601. doi: 10.1073/pnas.1708037114

8. Han JM, Periwal V. A mathematical model of calcium dynamics: Obesity and mitochondria-associated ER membranes. PLoS Comput Biol. 2019;15(8):e1006661. doi: 10.1371/journal.pcbi.1006661

9. Leger T, Jouve C, Patrac V, Batel V, Bouvier D, Sapin V, et al. A procedure to extract functional isolated mitochondria from small-sized human atrial samples. Application to obesity with a partial characterisation of the organelles. Free Radic Biol Med. 2020;153:71-79. doi: 10.1016/j.freeradbiomed.2020.04.006

10. Zheng Y, Yang N, Pang Y, Gong Y, Yang H, Ding W, et al. Mitochondria-associated regulation in adipose tissues and potential reagents for obesity intervention. Front Endocrinol (Lausanne). 2023;14:1132342. doi: 10.3389/fendo.2023.1132342

11. Poulsen SL, Moore SJ. Exercise affects fatty acid oxidation and lipid droplets in patients with type 2 diabetes. J Physiol. 2024;602(1):11-12. doi: 10.1113/JP285041

12. Cao X, Gao T, Lv F, Wang Y, Li B, Wang X. ROS-triggered and macrophage-targeted micelles modulate mitochondria function and polarization in obesity. Nanotechnology 2024;35(47). doi: 10.1088/1361-6528/ad7034
